# Accuracy of magnetic resonance spectroscopy in distinction between radiation necrosis and recurrence of brain tumors

**Published:** 2015-01-05

**Authors:** Mousa Reza Anbarloui, Seyed Mohammad Ghodsi, Alireza Khoshnevisan, Masoud Khadivi, Sina Abdollahzadeh, Ahmad Aoude, Soheil Naderi, Zeynab Najafi, Morteza Faghih-Jouibari

**Affiliations:** 1 Department of Neurosurgery, School of Medicine, Shariati Hospital, Tehran University of Medical Sciences, Tehran, Iran; 2 Department of Pediatrics, School of Medicine AND Children’s Medical Center, Tehran University of Medical Sciences, Tehran, Iran

**Keywords:** Magnetic Resonance Spectroscopy, Tumor Recurrence, Radiation Necrosis

## Abstract

**Background: **Distinction between radiation necrosis and recurrence of intraparenchymal tumors is necessary to select the appropriate treatment, but it is often difficult based on imaging features alone. We developed an algorithm for analyzing magnetic resonance spectroscopy (MRS) findings and studied its accuracy in differentiation between radiation necrosis and tumor recurrence.

**Methods:** Thirty-three patients with a history of intraparenchymal brain tumor resection and radiotherapy, which had developed new enhancing lesion were evaluated by MRS and subsequently underwent reoperation. Lesions with Choline (Cho)/N-acetyl aspartate (NAA) > 1.8 or Cho/Lipid > 1 were considered as tumor recurrence and the remaining as radiation necrosis. Finally, pre-perative MRS diagnoses were compared with histopathological report.

**Results: **The histological diagnosis was recurrence in 25 patients and necrosis in 8 patients. Mean Cho/NAA in recurrent tumors was 2.72, but it was 1.46 in radiation necrosis (P < 0.01). Furthermore, Cho/Lipid was significantly higher in recurrent tumors (P < 0.01) with the mean of 2.78 in recurrent tumors and 0.6 in radiation necrosis. Sensitivity, specificity, and diagnostic accuracy of the algorithm for detecting tumor recurrence were 84%, 75% and 81%, respectively.

**Conclusion: **MRS is a safe and informative tool for differentiating between tumor recurrence and radiation necrosis.

## Introduction

More than half of all brain tumors are intraparenchymal.^1^ Surgery is the main treatment for such tumors but microscopic spread of neoplastic cells and adjacency to eloquent areas, frequently prevents total tumor removal. Therefore, radiotherapy is necessary as an adjuvant treatment for better local control of in most patients.

A delayed complication of radiotherapy is radiation necrosis. It is a long-term complication that occurs 6 months to decades after radiation treatment. Pathologic changes include endothelial thickening of small arteries, lymphocyte and macrophage infiltration, hyalinization, fibrinoid deposition, thrombosis, and finally luminal occlusion. The vascular endothelial injuries cause damage to oligodendroglia. As a result, white matter tissue is often affected more than gray matter tissue.^2^ Radiation necrosis causes symptoms such as headache, seizure, mental changes and other neurological deficits.^3^ Therefore, it can mimic tumor recurrence clinically, but its management and prognosis are different.^4^ Furthermore, discriminating radiation necrosis from recurrent intraparenchymal neoplasm by imaging can be challenging because both can have regions with avid uptake of contrast material on T1-weighted images and can cause mass effect with local edema. Therefore, it seems necessary to seek other imaging techniques to differentiate between them. In this regard, numerous studies have investigated the capabilities of new imaging techniques such as positron emission tomography (PET),^5^^-^^7^ single photon emission computed tomography (SPECT),^8^^-^^10^ diffusion-weighted imaging (DWI) and magnetic resonance spectroscopy (MRS).^11^^,^^12^

MRS is a noninvasive technique, which shows metabolite profile of the brain.^13^ It does not use harmful radionuclide tracers and is available in most MRI centers. Spectroscopic characteristics of radiation necrosis and recurrent tumors such as astrocytoma, oligodendroglioma and metastasis have been explained in the literature.^14^^-^^17^ In this prospective study, we propose a model to analyze spectroscopic findings and investigate its accuracy in discrimination between radiation necrosis and recurrence of intraparenchymal tumors.

## Materials and Methods

Within a period of 28 months, a total of 33 patients (20 males and 13 females) with history of intraparenchymal brain tumor resection and subsequent radiotherapy (ranged from 55 to 65 Gy) were enrolled in the study. All the patients had symptoms related to a new parenchymal enhancing lesion in the vicinity of the original treated tumors. Thirteen patients had glioblastoma, six had low-grade astrocytoma, five had anaplastic astrocytoma, two had oligodenroglioma, five had metastasis and two had medulloblastoma (Table 1). All the patients were evaluated by MRS and subsequently underwent reoperation. Finally, preoperative MRS diagnoses were compared with histopathological report. After explaining the details of the procedures to the patients, Informed consent was obtained.

MRS was performed during conventional MR image acquisition with a 1.5-T MR unit, Magnetom, Vision (Siemens, Erlangen, Germany). The single-voxel technique was employed using a standard voxel volume of 8.0 cm^3^ (2.0 × 2.0 × 2.0 cm) or at least 3.37 cm^3^ (1.5 × 1.5 × 1.5 cm) applied in smaller lesions. The parameters utilized for acquisition were the Press technique using a TE of 144 ms. Shimming was automatically performed, followed by chemically selected saturation for water suppression. When possible, signal contamination from fat tissue in the skull and skull base was avoided. Voxels were positioned on T2-weighted images of the lesions, and adjusted spectra were always acquired before the administration of gadolinium-diethylenetriamine penta-acetic acid (Gd-DTPA). T1-weighted MR imaging with Gd-DTPA was then obtained to confirm the spatial relationship between the spectroscopic voxel and the enhanced lesion. Patients younger than 10 years of age were sedated with halothane and nitrous oxide to obtain motion-free examinations. Metabolites were always shown, on the x-axis in parts per million (ppm) and on the y-axis, by the height of the metabolite peaks in an expressed scale at an arbitrary intensity. The metabolites studied were Choline (Cho) which appears at 3.22 ppm, N-acetyl aspartate (NAA) at 2.01 ppm and lipid at 0.8-1.3 ppm.

**Figure 1:**
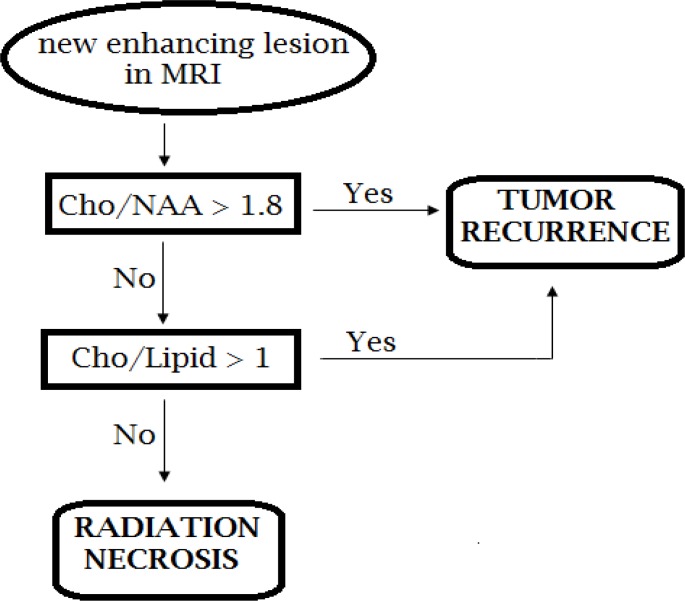
The algorithm for discrimination between tumor recurrences from radiation necrosis according to spectroscopic data

MRI: Magnetic resonance spectroscopy; Cho: Choline; NAA: N-acetyl aspartate

MRS data were evaluated using a commercial program (Luise; Siemens) available at the Magnetom Vision scanner. Signal intensity ratios Cho/NAA and Cho/Lipid were analyzed for the lesions. According to previous studies,^15^^,^^18^ we used cut-off value of 1.8 for Cho/NAA and 1 for Cho/Lipid*. *Cho/NAA > 1.8 and Cho/Lipid > 1, were considered as an indicator of tumor recurrence. Also, we proposed an algorithm shown in figure 1 which uses both Cho/NAA and Cho/Lipid ratios.

Preparation of biopsy specimens included neutral formalin fixation and paraffin embedding. Histological sections were stained with hematoxylin and eosin and examined with a light microscope. Tissue sections were analyzed for the presence of tumor and radiation necrosis.

## Results

**Figure 2:**
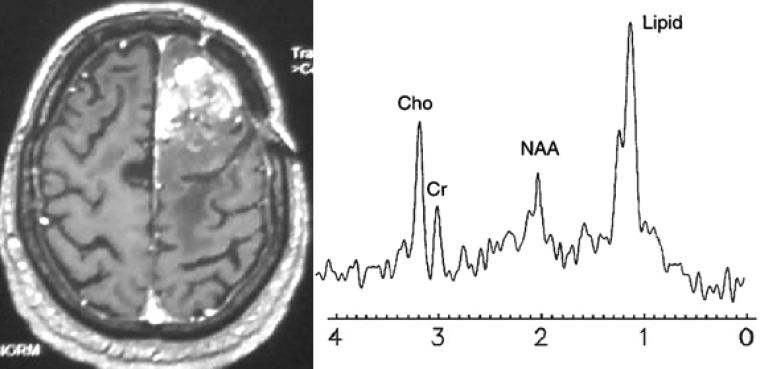
54-year-old woman after surgical resection and radiation for left frontal glioblastoma.(case 22) (a) Axial T1-weighted image after contrast administration shows a new area of contrast enhancement in left frontal lobe. (b) Spectra showed prominent lipid peak, with slightly decreased choline (Cho)/N-acetyl aspartate ratio (1.5) and decreased Cho/lipid ratio (0.6), indicating radiation necrosis, which was confirmed at histopathology

**Figure 3:**
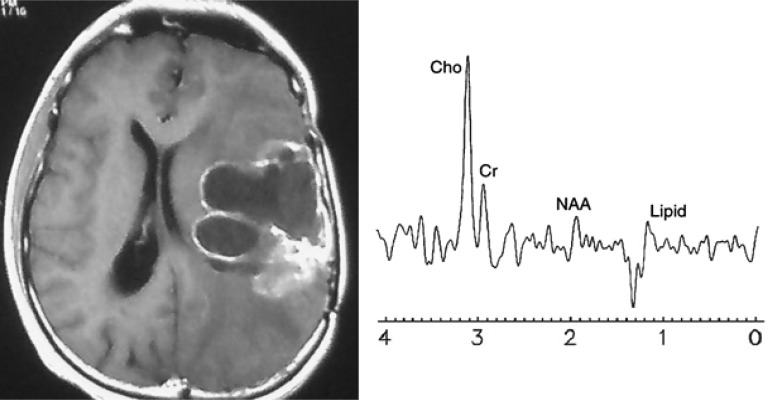
Magnetic resonance (MR) imaging and MR spectroscopy in a 44-year-old man with glioblastoma and history of surgery and radiotherapy (case 13). (a) Axial T1-weighted contrast-enhanced MR image, shows a left temporal enhancing lesion with mass effect. There is a solid enhancement in posterior part and peripheral enhancement in other areas, suggesting cyst or necrosis. (b) MR spectroscopic image, shows pathologic spectra with increased choline (Cho)/N-acetyl aspartate and Cho/lipid ratio (4.7 and 5.2, respectively), indicative of tumor recurrence. Histopathologic examination confirmed diagnosis

Surgical resection was performed in all the 33 patients. The histological diagnosis was recurrence in 25 patients and necrosis in 8 patients (Table 1). Typical examples of metabolic spectra obtained in a lesion consistent with radiation injury and in recurrent tumor are given in figures 2 and 3.

Mean Cho/NAA in radiation necrosis and recurrent tumors were 1.46 and 2.72 respectively, with a significant difference (P < 0.01). Also, Cho/Lipid was significantly higher in recurrent tumors (P < 0.01) with the mean of 0.6 in radiation necrosis and 2.78 in recurrent tumors.

The mean interval between completion of radiotherapy and MRS was longer in patients with radiation necrosis (16.3 months in contrast to 11.1 months), but the difference was not significant (P = 0.07).

MRS diagnosis using Cho/NAA value was different from diagnosis according to Cho/Lipid value, in eight patients (for example, in favor of radiation necrosis by Cho/NAA and tumor recurrence by Cho/Lipid)***. ***The final logistic regression model had an area under the receiver operating characteristic (ROC) curve of 85% using Cho/NAA and 89% using Cho/Lipid. Sensitivity, specificity, and diagnostic accuracy of Cho/NAA, Cho/Lipid and the proposed algorithm for detecting tumor recurrence, are shown in table 2. 

**Table 1:** Demographic data, primary tumor pathology, total radiation dose and interval to MRS, metabolic ratios, MRS diagnosis and histopathological diagnosis of patients

**Age**	**Gender**	**Primary pathology**	**Radiation Dose (Gy)**	**Interval to MRS (months)**	**Cho/NAA**	**Cho/Lipid**	**Diagnosis a according to algorithm**	**Histopathologic diagnosis**
32	F	LGA	56	9	2.1	0.8	T	T
28	M	LGA	56	24	1.5	2.9	T	T
18	F	LGA	60	9	2.4	0.6	T	T
33	M	LGA	56	13	2.1	3.5	T	T
41	M	LGA	60	33	1.0	0.3	R	R
21	F	LGA	60	12	3.1	2.0	T	R
55	M	AA	64	14	4.2	3.5	T	T
41	M	AA	60	8	1.5	1.8	T	T
37	M	AA	64	22	3.0	4.7	T	T
60	F	AA	64	8	2.5	2.2	T	T
54	F	AA	60	13	1.1	0.4	R	T
68	F	GBM	60	7	2.5	2.0	T	T
44	M	GBM	64	10	4.7	5.2	T	T
38	M	GBM	60	11	1.6	0.6	R	T
55	F	GBM	64	5	4.7	3.7	T	T
64	F	GBM	64	10	4.1	6.3	T	T
41	M	GBM	64	13	1.5	0.6	R	T
57	M	GBM	64	7	4.1	4.2	T	T
71	F	GBM	64	8	3.9	2.5	T	T
63	M	GBM	60	11	3.1	0.8	T	T
51	M	GBM	64	17	2.0	0.5	T	R
54	F	GBM	60	12	1.5	0.6	R	R
39	F	GBM	56	11	1.3	0.6	R	R
35	M	GBM	60	17	0.8	0.2	R	R
51	M	Oligo	60	33	1.3	1.8	T	T
44	M	Oligo	64	20	1.0	0.4	R	R
59	M	Met	60	5	3.3	2.1	T	T
55	F	Met	60	4	1.8	4.1	T	T
66	F	Met	64	6	1.5	3.9	T	T
59	M	Met	60	7	1.6	0.8	R	T
69	F	Met	60	9	1.0	0.2	R	R
6	F	Medulo	54	14	3.3	5.4	T	T
6	M	Medulo	54	8	4.6	4.1	T	T

T :Denotes tumor recurrence; R: Denotes radiation necrosis; MRS: Magnetic resonance spectroscopy; Cho: Choline; NAA: N-acetyl aspartate; LGA: Low-grade astrocytoma; AA: Anaplastic astrocytoma; GBM: Glioblastoma; Oligo: Oligodenroglioma; Met: Metastasis; Medulo: Medulloblastoma

**Table 2:** Sensitivity, specificity and diagnostic accuracy of proposed algorithm

**Classification function**	**Cho/NAA (%)**	**Cho/Lipid (%)**	**Algorithm (%)**
Sensitivity	73	87	84
Specificity	75	87	75
Diagnostic accuracy	69	75	81

Cho: Choline; NAA: N-acetyl aspartate

## Discussion

In patients with intraparenchymal brain tumors and history of surgery and radiotherapy, it is important to differentiate between radiation necrosis and tumor recurrence to choose appropriate treatment and predict prognosis. In both situations, conventional MRI usually shows enhancing lesion with or without mass effect, so it cannot be conclusive. New imaging techniques such as PET, SPECT, DWI and MRS have been studied extensively to analyze their capabilities in discriminating between radiation necrosis and tumor recurrence. MRS is an inexpensive imaging technique that does not use harmful radionuclide tracers and can be performed with conventional MRI at the same time. There are many studies in the literature explaining spectroscopic characteristics of radiation necrosis and tumor recurrence.

Spectroscopic changes in radiation necrosis include slight depression of NAA and variable changes in Cho and Cr. Also, it may show Lipid or Lactate peak reflecting cellular debris in the lesion.^15^ In contrast, recurrent neoplastic lesions show prominent elevation of Cho peak due to high turnover of cell membrane.^13^^,^^19^^-^^23^ In order to reliable distinction of tumor recurrence and radiation necrosis according to spectroscopic findings, different metabolites and their ratios have been studied and some cutoff values have been introduced.

Kimura et al. used Cho/Lip or Lac ratio and found that in cases of radiation necrosis, a high lipid-dominant peak was observed from the central non-enhanced region, along with a low Cho peak and a low NAA peak.^24^ Similar figures were found in another study by the same group in which the authors could differentiate ring-enhancing lesions as “space-occupying radiation necrosis” from ring-enhancing metastasis in all 6 cases by using MRS.^25^

Using multi-voxel MRS, Rock et al. claimed that a Cho/Cr ratio > 1.79 or a lipid and Lactate/Cho ratio < 0.75 has sevenfold increased odds of being pure tumor compared with pure necrosis.^26^ Weybright et al. reported that when cutoff values of 1.8 for either Cho/NAA or Cho/Cr were used, 27 of 28 patients (97%) were retrospectively correctly diagnosed.^18^ In another study using multi-voxel 3D MRS, the investigators found the Cho/NAA and Cho/Cr ratios to be significantly higher in recurrent tumor than in radiation injury, whereas the NAA/Cr ratios were lower in recurrent tumor than in radiation injury. When they used ROC analysis, the resulting sensitivity, specificity, and diagnostic accuracy of 3D MRS were 94.1%, 100%, and 96.2%, respectively, based on the cut-off values of 1.71 for Cho/Cr or 1.71 for Cho/NAA or both as tumor criteria.^27^ Smith et al. studied 33 patients retrospectively and concluded that an elevated Cho/NAA ratio correlated with evidence of tumor recurrence.^28^

In our study, Cho/NAA and Cho/Lipid were significantly different between tumor recurrence and radiation necrosis. According to previous studies, we used cut-off value of 1.8 for Cho/NAA and 1 for Cho/Lipid (Figures 2 and 3). Both ratios had an acceptable accuracy but combining them by using algorithm shown in figure 1 resulted in greater diagnostic accuracy than each ratio (Table 2). Area under the ROC curve was 85% for Cho/NAA and 89% for Cho/Lipid which showed excellent discrimination between tumor recurrence and radiation necrosis.

 There were 6 patients with wrong MRS diagnosis. In three patients with neoplastic lesion, spectroscopic diagnosis was radiation necrosis; and in two of them, the lesions had a large volume (> 50 cm^3^) and it is probable that the neoplastic portion of the lesion was not included in MRS voxel. Therefore, it seems necessary to examine all portions of large lesions.

The mean interval between completion of treatment and MRS was calculated in the two groups and it was 11.1 months in recurrent tumors and 16.3 months in radiation necrosis. Although the difference was not significant (P = 0.07), it may indicate that radiation necrosis is a long-term complication and it is uncommon in early stages, especially in 6 months after radiation therapy.

We believe that MRS has an acceptable accuracy in patients with an enhancing brain lesion and history of intraparenchymal tumor resection and radiation. Considering the noninvasiveness and availability of MRS, it is reasonable to perform MRS in these patients. Addition of other imaging techniques (such as DWI) to spectroscopic data may improve the accuracy.

## Conclusion

In patients with intraparenchymal brain tumor and history of tumor resection and radiation who have developed new enhancing lesion, MRS is a safe and informative tool for differentiating between tumor recurrence and radiation necrosis. It is necessary to examine different portions of the high volume lesions.

## Notes

**How to cite this article: **Anbarloui MR, Ghodsi SM, Khoshnevisan A, Khadivi M, Abdollahzadeh S, Aoude A, et al. Accuracy of magnetic resonance spectroscopy in distinction between radiation necrosis and recurrence of brain tumors. Iran J Neurol 2015; 14(1): 29-34.
